# Is HbA1c an ideal biomarker of well-controlled diabetes?

**DOI:** 10.1136/postgradmedj-2020-138756

**Published:** 2020-09-10

**Authors:** Georgia Kaiafa, Stavroula Veneti, George Polychronopoulos, Dimitrios Pilalas, Stylianos Daios, Ilias Kanellos, Triantafyllos Didangelos, Stamatina Pagoni, Christos Savopoulos

**Affiliations:** First Propedeutic Department of Internal Medicine, AHEPA University Hospital, Medical School, Aristotle University of Thessaloniki, Greece; First Propedeutic Department of Internal Medicine, AHEPA University Hospital, Medical School, Aristotle University of Thessaloniki, Greece; First Propedeutic Department of Internal Medicine, AHEPA University Hospital, Medical School, Aristotle University of Thessaloniki, Greece; First Propedeutic Department of Internal Medicine, AHEPA University Hospital, Medical School, Aristotle University of Thessaloniki, Greece; Internal Medicine Department, General Hospital of Serres, Greece; First Propedeutic Department of Internal Medicine, AHEPA University Hospital, Medical School, Aristotle University of Thessaloniki, Greece; First Propedeutic Department of Internal Medicine, AHEPA University Hospital, Medical School, Aristotle University of Thessaloniki, Greece; 3rd Department of Internal Medicine, "G. Gennimatas" General Hospital of Athens, Greece; Hellenic Society of Internal Medicine, Athens, Greece; First Propedeutic Department of Internal Medicine, AHEPA University Hospital, Medical School, Aristotle University of Thessaloniki, Greece

**Keywords:** Diabetes & endocrinology, General diabetes, Haematology, Anaemia

## Abstract

HbA1c is a biomarker with a central role in the diagnosis and follow-up of patients with diabetes, although not a perfect one. Common comorbidities encountered in patients with diabetes mellitus, such as renal insufficiency, high output states (iron deficiency anaemia, haemolytic anaemia, haemoglobinopathies and pregnancy) and intake of specific drugs could compromise the sensitivity and specificity of the biomarker. COVID-19 pandemic poses a pressing challenge for the diabetic population, since maintaining optimal blood glucose control is key to reduce morbidity and mortality rates. Alternative methods for diabetes management, such as fructosamine, glycosylated albumin and device-based continuous glucose monitoring, are discussed.

## 1. INTRODUCTION

Blood glucose regulation is a key factor for an effective immune response against infections and that is the main reason why diabetics have 4.4 times greater risk of systemic infection than non-diabetics.^1, 2, 3^ In the current SARS-CoV-2 setting optimal glucose control is of utmost importance in order to avoid excess morbidity and mortality and to optimise healthcare resource allocation. Furthermore, according to recent data, diabetic COVID-19 patients are at higher risk of severe pneumonia, hospitalisation and adverse clinical outcomes including increased mortality.^4, 5, 6^ Subsequently, accurate biomarkers for diagnosis and follow-up of diabetes mellitus (DM) are particularly relevant in the current setting. Glycosylated haemoglobin (HbA1c) has a central role in diabetes diagnosis and monitoring.

The aim of the current paper is to address the role and the limitations of HbA1c in specific patient populations.

## 2. ASSESSMENT OF HBA1C

Allen first recognised HbA1c in 1958 as component of human haemoglobin (Hb), while 10 years later Rahbar demonstrated the presence of an ‘abnormal’ Hb in diabetic patients. In 1970, the first application of HbA1c was made, followed by the appearance of the first available method to measure HbA1c levels. In 1993, the importance of HbA1c in the prognosis of DM related complications was demonstrated, and in 2010 HbA1c ≥6.5% was established as a diagnostic criterion for DM by the American Diabetes Association (ADA).^7^

It is well known that glycosylation of Hb is achieved by glucose entering the erythrocyte through the GLUT1 transporter and its connection to the amino acid valine which is located at the N-terminal of the beta chain of Hb. This procedure leads in the creation of a Schiff base resulting in the formation of a stable Amadori product, which is known as HbA1c.^8^

ΗbA1c is useful in the diagnosis, prevention and monitoring of DM, reflecting the impact of lifestyle and medication on glycaemic control over the past 3 months. Moreover, HbA1c levels between 5.7% and 6.4% correspond to people with pre-diabetes who are at higher risk of developing DM.^9^ Intensive glycaemic control and lower levels of HbA1c are followed by reduction of diabetic complications; at HbA1c <7%, there is a 76% reduction in the incidence of diabetic retinopathy, 54% of diabetic nephropathy, 60% of peripheral neuropathy and 35% of cardiovascular disease risk.^10^ On the other hand, lowering HbA1c levels is associated with higher risk of hypoglycaemia prevalence among patients with type 1 and type 2 diabetes. Several studies have demonstrated a negative relationship between HbA1c and hypoglycaemia in diabetic patients.^11  12^

Measurement of HbA1c should comply with international methods in the absence of conditions that may interfere with the results.^13^ There are two basic principles of HbA1c measurement: either with separation of HbA1c from other Hb fractions by chromatography and electrophoresis, or by targeting HbA1c as an antigen with immunochemistry. More specifically, there are four methods of measurement: (i) ion-exchange high-performance liquid chromatography (HPLC), (ii) boronate affinity HPLC, (iii) immunoassay and (iv) enzymatic assays. Affinity chromatography measures total HbA1c and is less affected by the presence of Hb variants, but does not separate HbA1c species and overestimates its values, while immunoassays are affected by high HbF values. In particular, at the end of the beta chain of HbF molecule, there is glycine instead of valine, which is acetylated thus contributing to an underestimation of HbA1c levels by almost 20%.^14^ As a result, it was considered necessary for HbA1c measurement methods to be standardised and so in 1996, the National Glycohemoglobin Standardization Program (NGSP) was introduced, which measures HbA1c in percentage and later in 2001 the International Federation of Clinical Chemistry and Laboratory Medicine (IFCC) which measures HbA1c in mmol HbA1c/mmol Hb and the estimated average glucose (eAG), which is not particularly used. IFCC reference values—due to their greater specificity—are 1.5–2% units below NGSP values, time consuming and not widely used in clinical practice.^8^ It is accepted that any change in HbA1c value by at least 0.5% in a laboratory certified by NGSP is considered statistically and clinically significant, while any change about 1% of HbA1c is associated with a ~30 mg/dL change in plasma glucose levels.^8^

In comparison with other methods of DM diagnosis, HbA1c measurement may have a higher cost but does not require fasting before measurement and it is not affected by stressful situations or the acute phase of the disease.^15^ If the sample cannot be measured immediately, it can be maintained safely either at 2–8°C for 4 weeks or at room temperature for 2 weeks.^16^

It has been established that patients with same HbA1c values may have true average glucose concentrations that differ by more than 60 mg/dL.^17^ A mathematical model that contributes to the improvement of the accuracy of average glucose estimation from HbA1c has recently been developed. This model is based on the combination of haemoglobin glycation and red blood cell kinetics with successive patient glucose measurements to extract patient-specific estimates of non-glycaemic determinants of HbA1c, including mean erythrocyte age.^17^

## 3. DIAGNOSTIC PITFALLS OF HBA1C

The data in the literature remain conflicting with regard to the establishment of the most suitable diagnostic test of DM. Although HbA1c has been recognised by few studies as equivalent or superior screening test compared with fasting plasma glucose (FPG) and as valuable as the oral glucose tolerance test (OGTT), there have been also studies with opposite results.^18^ Estimated glycaemic control with HbA1c alone may not be completely accurate in some diabetics and does not properly affect treatment decisions.^19^ In any case, the application of all three ways of glucose estimation contributes to the better identification of individuals who are likely to develop DM.

It is already known that HbA1c values fluctuate even in non-diabetic individuals (in healthy population only 50% of HbA1c fluctuations are affected by glucose levels).^20^ In particular, the HbA1c value may be affected by changes in the amount of glucose that penetrates the erythrocyte membrane, by a change in the rate of glycosylation or by a change in the erythrocyte’s lifespan. Any condition that prolongs or shortens the lifespan of erythrocytes or is associated with a reduced or increased rate of regeneration, exposes the erythrocytes for longer or shorter duration to the effect of glucose, resulting in an increased or decreased HbA1c value, respectively.^9^ Moreover, there is a large heterogeneity in the lifespan of red blood cells in haematologically healthy individuals. Older red blood cells have already been glycosylated to a greater extent than younger ones, while younger ones are more numerous and have an average half-life of about 30 days. Half of the result of a given HbA1c measurement is due to the contribution of erythrocyte glycosylation over the last 30 days, while glycosylation of red blood cells aged 90–120 days reflects only the 10% of the HbA1c value.^21^ Other factors that affect HbA1c in healthy population include age as there is a 0.1% increase in HbA1c for every 10 years, race (0.1–0.4% decrease in HbA1c has been observed in Caucasians compared with non-Caucasian populations for the same glucose levels) and pregnancy, because there is a reduced lifespan of erythrocytes from 120 to 90 days, with low HbA1c values in the second trimester, plateau at 20–24 weeks and increase in the third trimester.^1^

Improper glycaemic control reduces erythrocyte lifespan because the permeability of the erythrocyte membrane changes, the shape of red blood cells is distorted and the amount of sorbitol in red blood cells increases, which dramatically affects the activity of NA-K ATPase resulting in erythrocyte destruction. In addition, red blood cells of patients with DM have a higher phosphatidylserine content in the erythrocyte membrane and this contributes to their better recognition and destruction by the reticuloendothelial system.^22^

Several factors that could lead to false increase or false decrease in levels of HbA1C are summarised in online supplemental table 1.

### 3. 1. False increase

Conditions that prolong the lifespan of red blood cells such as iron deficiency anaemia, B_12_ and folic acid deficiency anaemia due to reduced erythrocyte proliferation or functional asplenia due to decreased destruction of blood cells could falsely increase HbA1c levels.^16^ Severe hypertriglyceridaemia (>1750 mg/dL), severe hyperbilirubinaemia (>20 mg/dL) and uraemia (due to carbaminohaemoglobin) also constitute commonly reported conditions associated with falsely elevated HbA1c. Alcohol intake due to the creation of a complex with acetaldehyde may cause false increase of HbA1c levels. Furthermore, several drugs and substances have been reported to interfere with HbA1c levels such as salicylates, opioids and lead poisoning.^16^

### 3. 2. False decrease

On the other hand, there are conditions that cause falsely decreased levels of HbA1c such as anaemia due to blood loss, or haemolytic anaemia by shortening the lifespan of erythrocytes, and splenomegaly as it leads to increased red cell turnover.^23^ Pregnancy also results in false decrease of HbA1c levels because of the decreased life span of the red blood cells. Moreover, the use of vitamin E, ribavirin, interferon A, cephalosporins, levofloxacin, penicillins, anti-inflammatory drugs and quinine (may cause haemolytic anaemia) has been associated with falsely decreased HbA1c levels.^16  23^

It is worth mentioning the controversial effect of vitamin C on HbA1c levels which is often used over the counter as component of vitamin supplements especially in the era of COVID-19 pandemic. Randomised clinical trials (RCTs) testing the effects of vitamin C on several biomarkers of glucose control such as HbA1c have reported variegated findings. A metaanalysis and systematic review by Ashor *et al* demonstrated that HbA1c concentration was not modified by vitamin C supplementation.^24^ Moreover, the data synthesis from five RCTs of vitamin C administration revealed a significant reduction in glucose levels without a significant effect on HbA1c levels.^25^ However, previously published metaanalysis reported a significant decrease in HbA1c levels.^26^

Furthermore, HbA1c is not reliable in patients suffering from homozygous haemoglobinopathy, while in heterozygous forms there is reliability depending on the measurement method. Presence of haemoglobinopathy should be suspected when there is a discrepancy between the HbA1c value and the patient’s daily glucose measurements, when HbA1c >15%, when there is a significant difference in HbA1c compared with previous values and when there is anaemia with abnormal red cell indices. In hereditary persistence of HbF, either in homozygous or in heterozygous form, falsely lower HbA1c values have been observed.^27^

Patients with end-stage renal disease present falsely decreased HbA1c values due to reduced proliferation rate and differentiation of erythrocyte precursor cells and ineffective response to endogenous erythropoietin (anaemia of chronic disease). Additional factors such as use of erythropoietin injections and uraemia could also modify HbA1c levels. In these patients, glycaemic control should be monitored with other methods except HbA1c, such as the measurement of fructosamine and glycosylated albumin (these methods estimate the glycaemia of the last 3 weeks because the half-life of albumin is 14–20 days).^28^ In diabetic haemodialysis patients, the measurement of glycosylated albumin is more reliable than the measurement of HbA1c, and in fact values of glycosylated albumin between 15.6% and 18.2% are considered to be associated with lower annual mortality. Moreover, it is known that in this group of patients the measurement of glycosylated albumin shows a much lower percentage of the burnt-out diabetes phenomenon .^29^ Nevertheless, diagnostic pitfalls in the measurements of fructosamine and glycosylated albumin may occur in cases of hypoproteinaemia, such as cirrhosis and nephrotic syndrome, and also in hyperlipidaemia.

## 4. ALTERNATIVE METHODS OF ESTIMATING GLYCAEMIA

Another method of estimating glycaemia over the last 48 hours to 2 weeks is to measure 1.5-anhydroglucytol (1.5-AG), which essentially reflects postprandial glycaemia. Limitations include renal, hepatic insufficiency and pregnancy.^30^

Finally, continuous glucose monitoring (CGM), which reflects glucose in the interstitial space and shows glycaemic levels of the last 3–7 days, is an additional way to measure glucose, especially for the treatment of high-risk diabetic patients, such as those with stroke or acute coronary syndrome, in which fasting glucose and oral glucose tolerance could provide safe information only 4 days after the acute episode, because during this time there is intense activation of the sympathetic nervous system and insulin resistance.^31  32^ CGM method emerges as a very valuable tool improving safety and effectiveness of diabetes treatment, reducing hypoglycaemia and decreasing glycaemic variability.^33^ More specifically time in range (TIR) of 70–180 mg/dL (3.9–10 mmol/L) derived from CGM devices has been proven as a significant index of glycaemia which reflects microvascular and macrovascular complications and it can supplement HbA1c outcomes.^34^

Although for several decades intensive glycaemic control has been the predominant target for the treatment of diabetic patients, studies have shown that achieving a HbA1c <7% was not associated with additional cardiovascular benefit, shifting the goal of DM treatment to reducing the risk of developing diabetic complications rather than achieving of lower glycaemic levels.^35^ Nevertheless, HbA1c remains unquestionably a very important method for assessing glycaemic control in diabetics (long-term glycaemic control and risk of developing complications), while the other methods (fructosamine, glycosylated albumin, 1.5-AG and CGM) are used to supplement the data, resulting from HbA1c measurements and in situations where HbA1c results are considered precarious.

In the absence of a formula to correct the discrepancies in the observed HbA1c values and the glycaemic status in specific patient populations, the physicians managing diabetes mellitus should follow an individualised approach to ensure optimal treatment. In the current pandemic milieu, uncontrolled diabetes has been associated with worse outcomes in COVID-19 patients.^36  37^ Thus, extra caution is warranted when assessing glycaemic control with HbA1c in patients with the aforementioned comorbidities, as a modifiable risk factor may go unchecked.

**Main messages**

1. HbA1c should be interpreted with caution in patients with frequently encountered comorbidities.
2. Chronic kidney disease, functional asplenia, iron,B12 and folic acid deficiency anaemia are among the conditions associated with falsely increased HbA1c levels.
3. Pregnancy and conditions such as splenomegaly, anaemia due to blood loss and haemolytic anaemia are associated with falsely decreased HbA1c levels.
4. Alternative biomarkers (fructosamine, glycosylated albumin, 1.5-anhydroglucytol) and methods such as continuous glucose monitoring may be more reliable in specific situations.

**Self-assessment questions**

1. **Which of the following factors could lead to falsely increased levels of HbA1c?**
  - Functional asplenia.
  - Splenomegaly.
  - Chronic alcohol intake.
  - Iron deficiency anaemia.
2. **Which of the following factors could lead to falsely decreased levels of HbA1c?**
  - Haemolytic anaemia.
  - Pregnancy.
  - Chronic kidney disease.
  - Β_12_ deficiency anaemia.
3. **Which of the following methods are the most suitable for estimating glycaemia in patients with acute stroke?**
  - HbA1c.
  - Fasting glucose.
  - Continuous glucose monitoring.
  - Oral glucose tolerance test.
4. **Which of the following methods are the most suitable for estimating glycaemia in patients with end-stage renal disease?**
  - HbA1c.
  - Glycosylated albumin.
  - Fructosamine.
  - Oral glucose tolerance test.
5. **Which of the following factors affect HbA1c values in non-diabetic individuals?**
  - Height.
  - Age.
  - Race.
  - Pregnancy.

**Current research questions**

1. Is HbA1c an ideal biomarker of well-controlled diabetes?
2. Is HbA1c measurement helpful in patients with many comorbidities?
3. Is uncontrolled diabetes associated with worse outcomes in COVID-19 patients?

**Key references**

1. d’Emden MC, Shaw JE, Jones GR, *et al.* Guidance concerning the use of glycated haemoglobin (HbA1c) for the diagnosis of diabetes mellitus. *Med J Aust* 2015;203:89–90. doi:10.5694/mja15.00041
2. Little RR, Roberts WLA. Review of variant hemoglobins interfering with hemoglobin A1c measurement. *J Diabetes Sci Technol* 2009;3:446–51. doi:10.1177/193229680900300307
3. Sacks DB. A1C versus glucose testing: a comparison. *Diabetes Care* 2011;34:518–23. doi:10.2337/dc10-1546
4. Radin MS. Pitfalls in hemoglobin A1c measurement: when results may be misleading. *J Gen Intern Med* 2014;29:388–94. doi:10.1007/s11606-013-2595-x
5. Beck RW, Connor CG, Mullen DM, *et al.* The fallacy of average: how using HbA1c alone to assess glycemic control can be misleading. *Diabetes Care* 2017;40:994–9. doi:10.2337/dc17-0636

**Answers**

1. a) **True**; b) **False**; c) **True**; d) **True**

2. a) **True**; b) **True**; c) **False**; d) **False**

3. a) **False**; b) **False**; c) **True**; d) **False**

4. a) **False**; b) **True**; c) **True**; d) **False**

5. a) **False**; b) **True**; c) **True**; d) **True**

## Supplementary Material

postgradmedj-97-380-DC1-inline-supplementary-material-1 Table 1. Conditions associated with limitations in HbA1c measurement interpretation
