# Supplementary material for: Is HbA1c an ideal biomarker of well-controlled diabetes?
Source: Postgrad Med J. 2020 Sep 10;97(1148):380–3. doi: 10.1136/postgradmedj-2020-138756 (PMC10016911; doi:10.1136/postgradmedj-2020-138756)
Supplement: postgradmedj-97-380-DC1-inline-supplementary-material-1 — Table 1. Conditions associated with limitations in HbA1c measurement interpretation [file postgradmedj-97-380-dc1-inline-supplementary-material-1.pdf]

Table 1.Conditions associated with limitations in HbA1c measurement interpretation

False relative increase	False relative decrease
Iron deficiency anemia<br>B12 or folic acid deficiency anemia	Anemia due to blood loss<br>Hemolytic anemia
Functional asplenia	Splenomegaly
Chronic Kidney Disease	Pregnancy
Severe metabolical abnormalities ((triglycerides >1750 mg/dl or bilirubin>20mg/dl)	Vitamin E intake
Chronic alcohol intake	Use of ribavirin, interferon A, cephalosporins, levofloxacin, penicillins, anti-inflammatory drugs and quinine
Chronic ingestion of salicylates and opioids	
Lead poisoning	
Variable effect in the case of hemoglobinopathies, vitamin C ingestion and blood transfusion	
